# Reliability of Rapid On‐Site Evaluation Achieved by Remote Sharing Systems (E‐ROSE) and AI Algorithms (AI‐ROSE) Compared With the Gold Standard in the Diagnosis of Lung Cancer

**DOI:** 10.1111/resp.70104

**Published:** 2025-08-11

**Authors:** Pasquale Tondo, Giuseppe Antonio Palmiotti, Giancarlo D'Alagni, Terence Campanino, Giulia Scioscia, Francesco Inglese, Renato Giua, Leonardo Monteleone, Maria Cristina Colanardi, Gianluca Libero Ciliberti, Armando Leone, Antonio Notaristefano, Ruggiero Torraco, Grazia Napoli, Grazia Marangi, Michele Pirrelli, Maria Pia Foschino Barbaro, Crescenzio Gallo, Donato Lacedonia

**Affiliations:** ^1^ Department of Medical and Surgical Sciences University of Foggia Foggia Italy; ^2^ Respiratory and Intensive Care Unit, Department of Specialistic Medicine University‐Hospital Polyclinic of Foggia Foggia Italy; ^3^ Pulmonology Unit ‘San Giuseppe Moscati’ Hospital, Local Health Authority of Taranto Taranto Italy; ^4^ PET/CT Diagnostic Unit ‘San Giuseppe Moscati’ Hospital, Local Health Authority of Taranto Taranto Italy; ^5^ Pathologic Anatomy Unit ‘Santissima Annunziata’ Hospital Taranto Italy; ^6^ Department of Clinical and Experimental Medicine University of Foggia Foggia Italy

**Keywords:** cytopathology, lung cancer, machine learning, ROSE, telehealth

## Abstract

**Background and Objective:**

In recent decades, artificial intelligence has seen significant development in various fields of medicine, including interventional pulmonology. The study aims to evaluate the diagnostic performance of innovative approaches to detect lung cancer on biopsy sample images (Rapid On‐Site Evaluation, ROSE) compared to the diagnostic gold standard.

**Methods:**

We conducted a multicentric study, comparing remote anatomopathological evaluation (E‐ROSE) and machine learning algorithms (AI‐ROSE) reliability in diagnosing lung cancer, evaluating 277 biopsy sample images, 25 of which were doubtful; to compare them with the definitive histological examination performed by the pathologist.

**Results:**

E‐ROSE achieved a diagnostic accuracy of 95.5%, with a sensitivity of 99.0% and specificity of 88.7%, including doubtful cases respectively 91.4%, 97.1%, and 81%. AI‐ROSE showed a sensitivity of 96.4% and a specificity of 78.9%, with an accuracy of 92.5%. Including the doubtful cases, the best model achieved an accuracy of 85%, sensitivity of 97.4%, and specificity of 75.4%. The discriminative ability of the tests was compared both for positive/negative cases, showing Area Under ROC Curve (AUC) of 93.9% for E‐ROSE and 87.6% for AI‐ROSE; while including doubtful, AUC was 89.1% for E‐ROSE and 86.4% for AI‐ROSE.

**Conclusions:**

The study suggests that the application of innovative methods such as E‐ROSE and AI‐ROSE could provide valuable support to interventional pulmonologists in the diagnostic process.

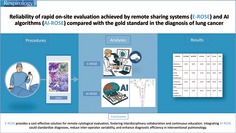

## Graphical abstract

The use of sharing technology (E‐ROSE) and machine learning models (AI‐ROSE) could facilitate reliable rapid on‐site evaluation (ROSE) of cytology images. E‐ROSE has demonstrated high sensitivity and accuracy; similarly, AI‐ROSE has shown good performance. These tools could optimise diagnosis in centres without dedicated cytopathologists.

## 1 Introduction

In recent years, interventional pulmonology has evolved rapidly with the adoption of innovative technologies that have improved the diagnosis and treatment of lung diseases [1]. Among these innovations, the integration of Rapid On‐Site Evaluation (ROSE) with telemedicine and artificial intelligence (AI) is an important step forward, offering new opportunities to optimise clinical effectiveness and accessibility of care.

ROSE [2] is a diagnostic technique that enables immediate cytologic evaluation of biopsy specimens during interventional procedures, such as Endobronchial Ultrasound‐Guided Transbronchial Needle Aspiration (EBUS‐TBNA) [3] and Transthoracic Needle Aspiration (TTNA) ultrasound [4]. This approach allows real‐time determination of the adequacy of collected specimens, reducing the need for repeat biopsies and thus improving procedural efficiency and patient experience. However, not all specialty centres have an experienced cytologist to perform ROSE, which is why many rely on specially trained technicians or pulmonologists [5].

In parallel, telemedicine has revolutionised health care, enabling remote consultation and supervision. The integration of telemedicine [6] with ROSE has the potential to extend the benefits of on‐site assessment to remote or less specialised healthcare facilities where the presence of an experienced cytopathologist is not always guaranteed. With advanced communication systems and secure digital platforms, biopsy specimens can be evaluated in real time by remote specialists, ensuring uniform and high diagnostic quality. A practical example is the use of secure messaging applications, such as WhatsApp, which, thanks to end‐to‐end encryption, allows images and diagnostic information to be shared securely (E‐ROSE).

The adoption of AI in clinical data analysis could also further improve the effectiveness of ROSE and telemedicine [7, 8]. Machine learning algorithms can be trained to analyse cytology specimens, assisting cytopathologists in identifying pathological cells and reducing the time needed to obtain an accurate diagnosis. AI offers the advantage of standardising assessments, reducing inter‐operative variability and increasing diagnostic accuracy.

Despite technological advances, the early diagnosis of lung neoplasms continues to present significant challenges due to poor and late symptomatology and still unoptimized screening programs [9]. We hypothesise that the synergistic combination of ROSE, telemedicine, and AI could not only improve the quality of care but also foster greater interdisciplinary collaboration and continuing education of health care providers. In addition, by reducing diagnosis time and optimising the use of healthcare resources, this integration could lead to decreased overall costs and improved clinical outcomes. Therefore, the aim of this study was to analyse the results of innovative methods such as the application of E‐ROSE and AI compared with definitive cytohistological examination in order to evaluate their usefulness and reliability in clinical practice.

## 2 Methods

### 2.1 Study Design

A multicentric study was conducted on anonymized ROSE‐valid biopsy specimen images obtained from patients evaluated by a multidisciplinary Thoracic Oncology team for suspected primary or secondary pulmonary neoplasm. Patients were selected on the basis of suspicious lesions evidenced by total body CT and/or PET‐CT, and deemed to be candidates for cytohistological investigation between June 2023 and December 2024.

The images obtained by ROSE, that is, sampled by an experienced interventional pulmonologist, were shared anonymously via encrypted platforms to an anatomic pathologist (E‐ROSE) for evaluation of sample adequacy and positivity. Subsequently, the same specimen images were analysed with artificial intelligence software (AI‐ROSE) to compare the results with those obtained via E‐ROSE.

The diagnostic gold standard used for validation was the final histological report, possibly supplemented with immunohistochemistry.

The Institute Ethics Committee provided a consent waiver, as this was a retrospective analysis of anonymised data. However, we obtained procedural consent from all the study participants.

### 2.2 Biological Sampling

#### 2.2.1 Inclusion and Exclusion Criteria

Images of slides from patients with exophytic or endophytic central lung lesions, mediastinal lesions, ilo‐mediastinal lymphadenopathies, peripheral lung nodules or masses with bronchus sign, subpleural lesions that could be evaluated ultrasound, preoperative mediastinal staging of lung carcinoma, and mediastinal re‐staging of lung carcinoma after treatment were included in the study. The choice of the method to be used followed the principle of least invasiveness and lowest risk to the patient.

Exclusion criteria were found to be: severe arrhythmias (patients at high cardiologic/anesthesiologic risk) or recent myocardial infarction or unstable angina, refractory hypoxaemia, severe plateletopenia and coagulation deficiency, lesions that could not be sampled by flexible bronchoscopy (peripheral lesions or absence of bronchus sign) or by transthoracic ultrasound‐guided biopsy (retro‐costal lesion or lesion not in contact with the pleura), as well as denial of consent.

#### 2.2.2 Sampling Procedure

The images evaluated in the study were obtained by different sampling methods: EBUS‐TBNA, EUS‐B‐FNA, echo‐guided transthoracic needle biopsy, and transbronchial biopsy.

Specifically, Endobronchial Ultrasound‐Guided Transbronchial Needle Aspiration (EBUS‐TBNA) [10, 11] is an endoscopic procedure used to obtain lymph node, bronchial, or mediastinal tissue samples from mediastinal structures. Endobronchial ultrasound (EBUS) allows real‐time, transbronchial needle aspiration (TBNA) sampling. The EBUS bronchoscope used was the Olympus BF‐UC190F bronchoscope. Sampling was implemented using 22 Gauge needles (40 mm in length), with capillary sampling and negative pressure syringe aspiration. Transthoracic echo‐guided needle biopsy is a procedure used to obtain tissue samples from lung lesions in contact with the pleura, from lymphadenopathy, or from the pleura itself. This technique uses ultrasound to precisely guide the needle to the area of interest, allowing real‐time sampling, reducing the risk of vascular injury or pneumothorax. The needle of choice has been 20 and 18 Gauge. Transbronchial biopsy is a procedure used to obtain tissue samples using a flexible bronchoscope with a radial miniprobe under fluoroscopic guidance along with biopsy instruments. An Olympus inspecting bronchoscope BF‐1H190 and an operating bronchoscope BF‐1TH190 were used. Forceps with alligator sockets or the valve were chosen as sampling instrumentation.

#### 2.2.3 Analysis of Biological Samples

##### 2.2.3.1 ROSE

Samples obtained by EBUS or TTNA were ejected from the needle, placed on a glove slide, and then smeared against a second slide. In the case of bronchial biopsy, a rolling of the specimen by scalpel on a glove slide was implemented instead. Following this, one slide was fixed with alcohol‐based fixative for off‐site evaluation, and a second slide was air‐dried and stained with the May‐Grünwald Giemsa (MGG) method for rapid on‐site evaluation. Using 3 numbered containers (1–3, and), the slide was initially passed in fixative (container 1), then in eosin (container 2), allowing staining of the cytoplasm and finally, in haematoxylin (container 3), allowing staining of nuclei. The number of passages within each container was 10 passages [12]. Once the stained slide was obtained, it was placed on a Leica ICC50W microscope, and using 10× and 20× magnifications, images of a microscopic field were obtained and projected onto the screen. The personnel assigned to perform ROSE were referable to the medical team, thus implementing a ROSE. At the end of the procedure, the stained and fixed slides, together with the cytoinclusion, were sent to the anatomy‐pathology department for histological diagnosis and mutational study. The final histological report, with possible mutational analysis, was considered the reference standard.

##### 2.2.3.2 E‐ROSE

A pulmonologist expert has identified the best slide field and has acquired static cytological images directly from the microscope and then transmitted them remotely to the pathologists. On average, 1 to 5 images per case were transmitted, focusing on the most representative areas without the dynamic control typical of direct microscopy or whole‐slide imaging. The images obtained were then shared via encrypted platforms, anonymously, with remote anatomic pathologist colleagues to assess appropriateness and diagnostic suspicion. The platforms used allowed messages, images, audio, or video files to be sent with good image sharpness, even in HD quality. In this way, an off‐site cytologic evaluation model was implemented remotely (E‐ROSE). The anatomo‐pathologist involved in this study has over 10 years of experience in lung cytopathology and assessed the images, making their opinion in real‐time by defining the image: positive, doubtful, or negative. The morphological criteria used in the evaluation are those of the Pulmonary Pathology Society [12]. In doubtful cases, an additional one to a maximum five images of the same specimen were requested and re‐examined until a diagnostic consensus was reached. The pathologist contributed both to the diagnostic classification and to the refinement of the training set for the artificial intelligence.

### 2.3 Data Analysis

#### 2.3.1 Statistics

E‐ROSE and AI‐ROSE test results were compared with the diagnostic gold standard (definitive cytohistological examination) to calculate sensitivity, specificity, and accuracy. Sensitivity was defined as the ratio of the number of true positives to the sum of true positives and false negatives. Specificity was calculated as the ratio of the number of true negatives to the sum of true negatives and false positives. Accuracy was determined by dividing the sum of true positives and true negatives by the total number of cases analysed, including true positives, true negatives, false positives, and false negatives.

The sensitivity and specificity of the two methods were compared by the area under the ROC curve (AUC) to assess the discriminative ability of the tests. All statistical analyses were performed using Python software version 3.11.8.

#### 2.3.2 AI‐ROSE

**FIGURE 1:**
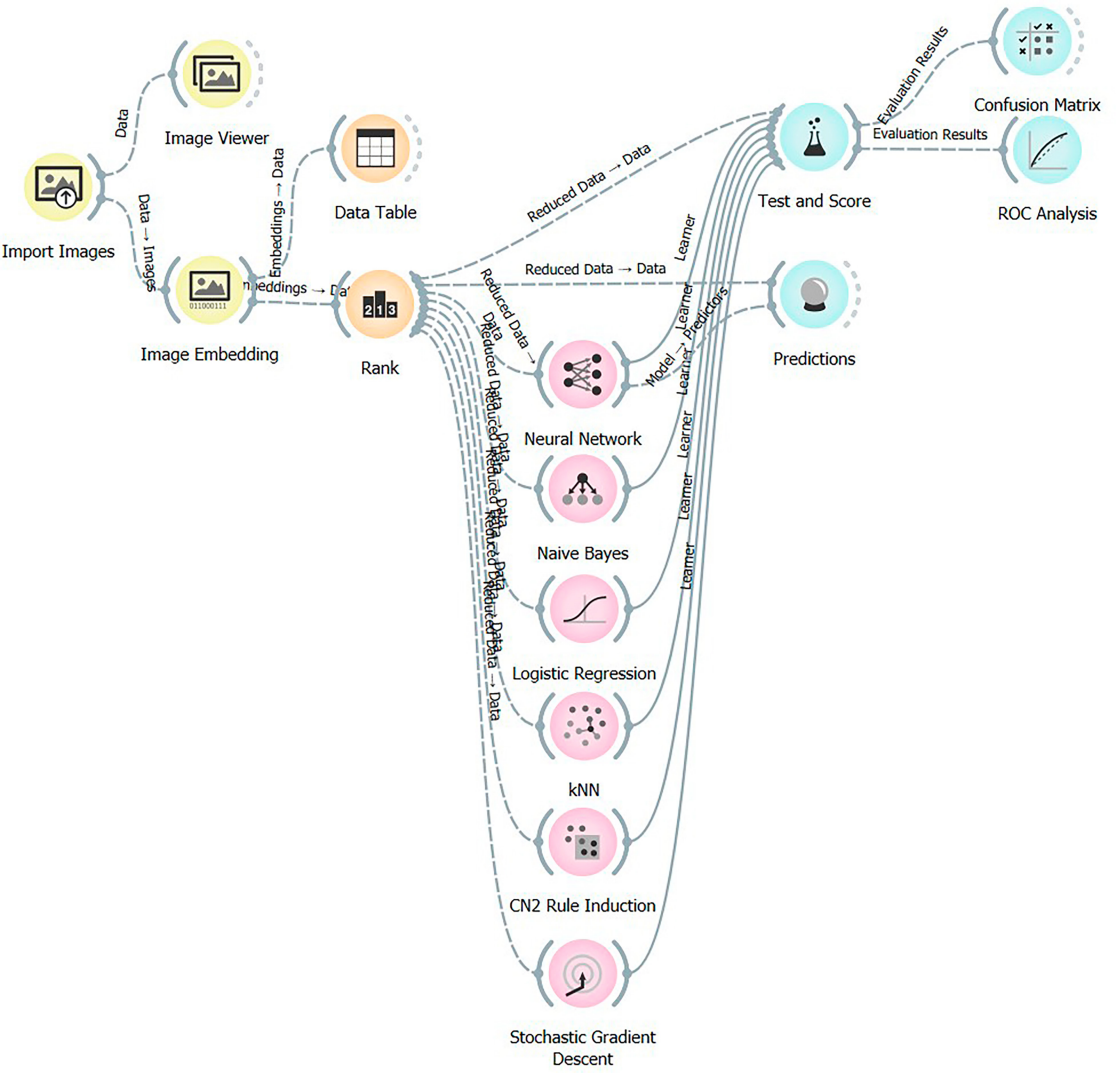
Image analysis steps to evaluate AI diagnostic yield using Orange software (read more detailed description in methods).

Orange 3.38.1 data‐mining software was used to analyse the images (Figure 1), which were divided into three categories (195 positive slides, 57 negative slides and 25 doubtful slides). Two types of analysis were conducted: the first considered only positive and negative slides (*n* = 252 images, including 195 positive and 57 negative), while the second included doubtful ones (*n* = 277 images including 195 positive, 57 negative and 25 doubtful). The images, after being imported and pre‐processed using the ‘Image Embedding’ widget, were processed with Google's ‘Inception v3’ model trained on ImageNet, which extracted 2048 features. To reduce the dimensionality of the data and select the most relevant variables, FCBF (Fast Correlation‐Based Filter) was applied, identifying 44 significant features in the first analysis and 55 in the second. Next, for image classification, several Machine Learning algorithms, including Neural Network (model composed of three hidden layers with 29, 58 and 58 neurons, ReLU activation and Solver Adam), Naive Bayes, Logistic Regression, kNN, CN2 Rule Induction, and SGD, were tested and validated by 10‐fold cross validation.

## 3 Results

**FIGURE 2:**
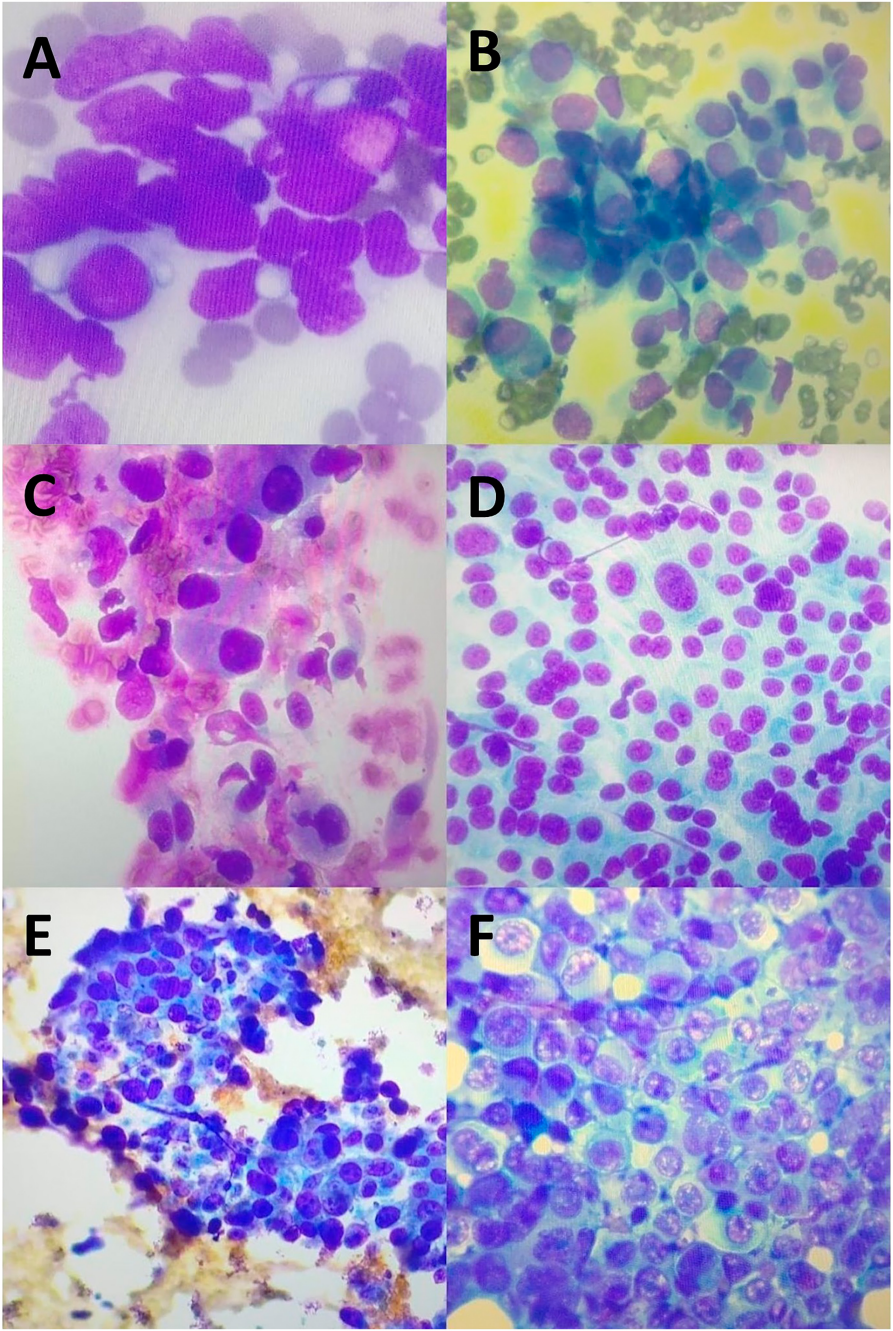
Slide collection. Resolution ×100. (A) Small cell lung cancer (SCLC); (B) pleural mesothelioma; (C) adenocarcinoma; (D) carcinoid tumour; (E) squamous cell carcinoma; (F) metastasis.

A total of 277 slide images were collected (Figure 2) from EBUS‐TBNA, transthoracic echo‐guided needle biopsy, and transbronchial biopsy procedures.

The diagnostic yield of the methodologies used (E‐ROSE and AI‐ROSE) is summarised in Table 1. Overall, analysis of the diagnostic performance of E‐ROSE responses showed a sensitivity of 99.0% and specificity of 88.7%, relative to the definitive histologic diagnosis on specimens classified as positive or negative. The overall diagnostic accuracy was 95.5%. A further analysis was also conducted, including doubtful cases, which showed a sensitivity of 97.1% and specificity of 81.0%, again with respect to the definitive histological diagnosis. In this re‐evaluation, the diagnostic accuracy was 91.4%.

**TABLE 1:** Diagnostic performance of E‐ROSE and AI‐ROSE.

Methods	Sensitivity	Specificity	Accuracy	AUC	F1 score	MCC
E‐ROSE (only positive/negative cases)	99.0%	88.7%	95.5%	93.9%	97.1%	90.7%
E‐ROSE (including doubtful cases)	97.1%	81.0%	91.4%	89.1%	91.0%	75.0%
AI‐ROSE (only positive/negative cases)	96.4%	78.9%	92.5%	94.8%	92.3%	77.9%
AI‐ROSE (Including Doubtful Cases)	97.4%	75.4%	85.2%	82.3%	83.5%	65.0%

Abbreviations: AUC, area under ROC curve; MCC, Matthew correlation coefficient.

As for AI‐ROSE, in the first analysis (positive and negative slides only), the neural network showed the best performance, with an AUC of 94.8%, Computed Accuracy (CA) of 92.5%, F1 of 92.3%, and Matthew correlation coefficient (MCC) of 77.9%. The performance of the neural network detects a sensitivity of 96.4% and a specificity of 78.9%. In the second analysis, which also included doubtful slides, the best model was found to be kNN with k = 5 and Euclidean metric, achieving an AUC of 82.3%, CA of 85.2%, F1 of 83.5%, and MCC of 65.0%. The performance of the kNN model showed a sensitivity on negative cases of 97.4% and a specificity of 75.4%. The results obtained show that the feature selection approach and the choice of predictive model have a significant impact on classification performance, with the neural network being more effective in distinguishing between positive and negative slides, while kNN performed better in classification including doubtful cases.

**FIGURE 3:**
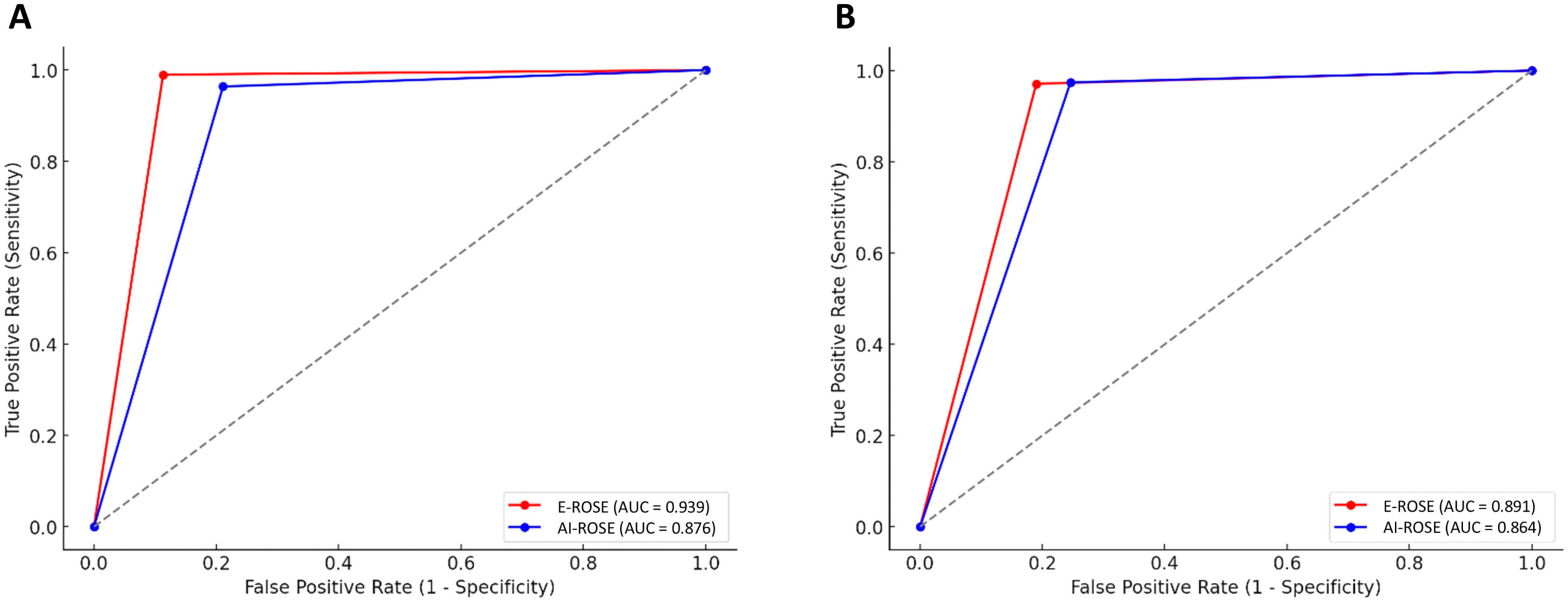
Area under the ROC curve (AUC) compared for both approaches, E‐ROSE and AI‐ROSE, both for positive and negative cases only (A), and including doubtful cases (B).

The discriminative ability of the tests was compared both for positive/negative cases only (E‐ROSE AUC = 93.9%, AI‐ROSE AUC = 87.6%) and including doubtful cases as well (E‐ROSE AUC = 89.1%, AI‐ROSE AUC = 86.4%); as shown in Figure 3.

## 4 Discussion

The present study suggests that the implementation of a clinical‐instrumental routine by applying innovative methods in clinical practice could help optimise the resources available to us in the diagnosis of lung cancer even in centres that do not have a dedicated anatomo‐pathologist available. In fact, this study stems from the widespread need in clinical practice to fill the lack of a dedicated anatomo‐pathologist during Interventional Pulmonology sessions to perform ROSE. Another important finding is that the time spent by the anatomo‐pathologist to read the slides is very small compared to the duration of the procedure, considering also the preparation and sedation of the patient. It follows that having an anatomo‐pathologist available for the entire duration of the procedure would not represent an optimal use of specialist resources, as their direct involvement is required only during specific, limited phases. In some cases, such as ours, the Anatomic Pathology units are located in facilities separate from those used for Interventional Pulmonology, which further complicates the availability of a dedicated pathologist for each procedure. Therefore, the application of systems such as E‐ROSE or AI‐ROSE contributes to a more efficient organisation, facilitates collaboration across distances, and can help optimise the allocation of healthcare resources.

To date, in centres where the anatomo‐pathologist is not available, the latter is replaced by an appropriately trained pulmonologist. However, there are discordant values on concordance between ROSE and definitive cytologic examination, with sensitivity ranging from 65% as in the study of Damaraju V et al. [2] to 91% as in the study of Bonifazi M. et al. [13]. In addition, training a pulmonologist to read ROSE also requires time and resources. The results of our study show that real‐time exchange of information and images (E‐ROSE) is a viable alternative to the physical presence of an anatomic‐pathologist in the room, with overall sensitivity and specificity values of 97% and 81%, respectively, and accuracy of 91.4%. These values increase by excluding doubtful cases, with sensitivity and specificity of 99% and 88.7%, respectively. Our results are in line with the study by Damaraju [2] et al., in which they show that the sensitivity of E‐ROSE was 92%. In our study, the same images sent via WhatsApp were also analysed by machine learning algorithms, with the aim of assessing the concordance between E‐ROSE and AI‐ROSE. The overall results, including doubtful cases, of sensitivity and specificity of AI‐ROSE were 97.4% and 75.4%, respectively, with a CA of 85.2%. As with E‐ROSE, the results of AI‐ROSE analysis are also slightly different without the doubtful cases, with sensitivity and specificity of 96.4% and 78.9%, respectively; although in this case, the results are overlapping.

Several studies have evaluated the performance of AI platforms in ROSE during EBUS‐TBNA, yielding promising results [14, 15]. For example, in the DEBUT study [14], it was demonstrated that their AI model could accurately classify EBUS‐TBNA ROSE slides as adequate or inadequate with high sensitivity (95.12%) and specificity (93.33%), using a dataset of 375 images. In the study by Shuang Yan et al., the AI system for ROSE showed promising performance in lung cancer diagnosis based on ROSE cytological images, achieving an accuracy of 89.61% and 87.59%, and a sensitivity of 90.57% and 94.90% in the internal and external test datasets, respectively. These findings are consistent with our results. In all these studies, as in ours, the assessment by an expert pathologist outperformed AI‐ROSE. However, their AI system achieved higher diagnostic performance compared to junior and intermediate‐level endoscopists.

Our study differs from the works of Asfahan et al. [14] and Yan et al. [15] in its application‐oriented approach, focusing on resource optimisation in clinical practice. While previous studies primarily explored the use of AI for cytological image classification, we directly compared the effectiveness of E‐ROSE and AI‐ROSE, evaluating their reliability against the gold standard histological diagnosis. This comparison allows us to assess both the diagnostic performance of AI and the impact of telemedicine in centres lacking dedicated pathologists.

Overall, the methods used in our study (E‐ROSE and AI‐ROSE) achieved comparable diagnostic performance, as confirmed by AUC values, demonstrating the reliability of both techniques in distinguishing between positive and negative samples, supporting their clinical applicability. The implications of these findings could be significant. First, pathologists could continue their laboratory activities while simultaneously providing remote support for ROSE. In settings lacking a pathology unit, AI integration represents a promising strategy for enhancing real‐time diagnostics. Although times were not systematically recorded, based on repeated clinical experience we can estimate that AI‐ROSE provided image classification in approximately 30 s, while E‐ROSE required between 1 and 3 min depending on image transmission time and pathologist availability. These time differences may be relevant in settings where rapid feedback is required. Additionally, the ability to capture, store, and share diagnostic images on a digital platform facilitates more effective interdisciplinary collaboration between pulmonologists and pathologists, positively impacting procedural outcomes. This approach could also support the training of specialists, granting them access to a vast database of high‐quality diagnostic images via mobile devices. From an economic perspective, this solution emerges as a more sustainable alternative to other telemedicine methods, helping to reduce costs without compromising diagnostic accuracy and reliability. Numerous studies have demonstrated that the adoption of ROSE during EBUS‐TBNA can reduce the number of needle passes required to obtain a diagnostic sample, improve procedural efficiency, and potentially lower overall costs. Specifically, according to Bott et al. (2015), the use of telecytopathology (AI‐ROSE) resulted in a significant reduction in needle passes, slides prepared, and average time to result confirmation compared to the conventional approach [16]. The direct material costs per procedure result equivalent between the ROSE and AI‐ROSE (about $888). The telepathology system becomes economically advantageous when monthly case volumes exceed certain thresholds [17].

However, this study presents some limitations. The analysis was conducted in a limited number of centres, involving pathologists and pulmonologists with varying levels of experience and learning curves, which could influence diagnostic accuracy. Furthermore, performing E‐ROSE requires an experienced pulmonologist in the endoscopy suite to select and capture images, a process that, although requiring less training than ROSE, introduces an additional variability factor related to operator expertise. A further limitation of E‐ROSE is its exclusive reliance on static image transmission, which prevents the pathologist from dynamically exploring the entire slide, thereby reducing the ability to perform a detailed analysis compared to direct microscopic evaluation. Another potential limitation is the sampling bias related to the manual selection of static cytological images. This introduces a degree of subjectivity and may omit diagnostically relevant areas, especially in samples with low cellularity or heterogeneous morphology. But we included ‘doubtful’ cases, those with ambiguous or low‐quality material, to assess the diagnostic robustness of both human interpretation and AI‐based systems. The inclusion of these challenging cases aimed to replicate real‐world clinical variability and to test model performance under suboptimal conditions. Image quality may also be affected by technical factors such as focus and lighting, which can influence diagnostic precision. Another limitation is the absence of an intra‐observer concordance analysis for the pathologist across different sample types. While such an analysis (e.g., using k‐statistics) could provide insight into interpretative consistency, it was not feasible in this study due to the predominance of EBUS‐TBNA samples and the cytological nature of all included specimens. Given the relative homogeneity of the material, we believe that the risk of interpretative variability is limited. Nevertheless, future studies involving a more diverse range of sample types may benefit from assessing intra‐observer variability. Regarding AI‐ROSE, its primary limitation is the need for a large dataset to refine the accuracy of the predictive model. The analysis was conducted on a relatively limited number of slides, and although the results are promising, large‐scale validation would be necessary to ensure greater reliability. Furthermore, AI may struggle to interpret cytological samples of suboptimal quality or with atypical morphological characteristics, making it less effective in complex or ambiguous cases compared to human judgement.

In conclusion, E‐ROSE remains a valuable low‐cost tool that can compensate for the absence of an on‐site pathologist in endoscopy suites, ensuring accurate remote cytological evaluation. Beyond its clinical applicability, this method facilitates interdisciplinary collaboration between pulmonologists and pathologists, supporting continuous education through an intuitive and easily accessible platform. On the other hand, AI‐ROSE represents an innovative option with the potential to actively assist interventional pulmonologists in real‐time cytological diagnosis. The integration of artificial intelligence could help reduce inter‐operator variability, improve diagnostic standardisation, and optimise reporting times. However, for AI‐ROSE to be widely implemented, further refinement of predictive models and extensive validation using larger and more diverse datasets are required.

Looking ahead, the combined adoption of E‐ROSE and AI‐ROSE could represent an optimal strategy to enhance diagnostic efficiency in interventional pulmonology, providing a more accessible, rapid, and standardised approach to cytological evaluation.

## Author Contributions

**Pasquale Tondo:** conceptualization (equal), formal analysis (equal), investigation (equal), methodology (equal), project administration (equal), supervision (equal), visualization (equal), writing – original draft (equal), writing – review and editing (equal). **Giuseppe Antonio Palmiotti:** conceptualization (equal), investigation (equal), methodology (equal), project administration (equal), resources (equal), supervision (equal), validation (equal), writing – original draft (equal), writing – review and editing (equal). **Giancarlo D'Alagni:** resources (equal), supervision (equal). **Terence Campanino:** data curation (equal), formal analysis (supporting), methodology (supporting), resources (equal), visualization (equal), writing – original draft (equal), writing – review and editing (equal). **Giulia Scioscia:** resources (equal). **Francesco Inglese:** resources (equal). **Renato Giua:** resources (equal). **Leonardo Monteleone:** data curation (equal), resources (equal), visualization (equal), writing – original draft (equal). **Maria Cristina Colanardi:** resources (equal). **Gianluca Libero Ciliberti:** resources (equal). **Armando Leone:** resources (equal). **Antonio Notaristefano:** resources (equal). **Ruggiero Torraco:** data curation (equal), resources (equal). **Grazia Napoli:** resources (equal). **Grazia Marangi:** resources (equal). **Michele Pirrelli:** resources (equal). **Maria Pia Foschino Barbaro:** resources (equal). **Crescenzio Gallo:** conceptualization (equal), formal analysis (equal), methodology (equal), software (equal). **Donato Lacedonia:** conceptualization (equal), formal analysis (equal), investigation (equal), methodology (equal), project administration (equal), supervision (equal), writing – original draft (equal), writing – review and editing (equal).

## Ethics Statement

The Institute Ethics Committee provided a consent waiver, as this was a retrospective analysis of anonymised data.

## Conflicts of Interest

The authors declare no conflicts of interest.

## Data Availability

The datasets used and/or analysed during the current study are available from the corresponding author on reasonable request.
